# Hydrogen Sulfide Regulates the Colonic Motility by Inhibiting Both L-Type Calcium Channels and BK_Ca_ Channels in Smooth Muscle Cells of Rat Colon

**DOI:** 10.1371/journal.pone.0121331

**Published:** 2015-03-26

**Authors:** Xiaojing Quan, Hesheng Luo, Yin Liu, Hong Xia, Wei Chen, Qincai Tang

**Affiliations:** 1 Department of Gastroenterology, Renmin Hospital of Wuhan University, Wuhan, China; 2 Department of Gastroenterology, the Affiliated Hospital of Guilin Medical College, Guilin, China; 3 Key Laboratory of Hubei Province for Digestive System Diseases, Wuhan, China; University of Texas Medical Branch, UNITED STATES

## Abstract

**Objective:**

To examine the hypothesis that hydrogen sulfide (H_2_S) regulates the colonic motility by modulating both L-type voltage-dependent calcium channels and large conductance Ca^2+^-activated K^+^ (BK_Ca_) channels.

**Methods:**

Immunohistochemistry was performed on rat colonic samples to investigate the localization of the H_2_S-producing enzymes cystathionine-β-synthase (CBS) and cystathionine-γ-lyase (CSE). The contractions of proximal colonic smooth muscle were studied in an organ bath system. The whole-cell patch-clamp technique was used to record both L-type calcium currents (*I*_Ca,L_) and BK_Ca_ currents in colonic smooth muscle cells (SMCs) isolated from male Wistar rats.

**Results:**

Immunohistochemistry revealed the presence of CBS and CSE in mucosa, smooth muscle cells and myenteric neurons. The H_2_S donor NaHS inhibited spontaneous contractions of the longitudinal muscle and circular muscle strips in a dose-dependent manner, and the inhibitory effects were not blocked by tetrodotoxin. NaHS inhibited the peak *I*_Ca,L_ in colonic SMCs at a membrane potential of 0 mV. The current-voltage (I-V) relationship of L-type calcium channels was modified by NaHS, and the peak of the I-V curve was shifted to the right. NaHS (200μΜ) evoked a significant rightward shift of the steady-state activation curve and inhibited the inactivation of L-type calcium channels. Furthermore, NaHS reversibly decreased the peak *I*_Ca,L_ in a dose-dependent manner. Likewise, BK_Ca_ channels were significantly inhibited by NaHS, and the addition of NaHS caused a time- and dose-dependent reduction in the BK_Ca_ current.

**Conclusion:**

The relaxant effect of H_2_S on colonic muscle strips may be associated with the direct inhibition of H_2_S on L-type calcium channels. H_2_S may be involved in the regulation of calcium homeostasis in colonic SMCs of rat colon.

## Introduction

Hydrogen sulfide (H_2_S), well-known for its peculiar odor, is generated endogenously in rat colon from the substrate L-cysteine by the actions of two enzymes, cystathionine β-synthase (CBS) and cystathionine γ-lyase (CSE) [1–3]. In addition to nitric oxide (NO) and carbon monoxide (CO), H_2_S has been identified as the third endogenous signaling gasotransmitter [4]. Functionally, H_2_S has been implicated in several physiological processes in the gut, including gastrointestinal (GI) motility [5,6], secretion [7], and neuromodulation [8]. There is growing evidence that H_2_S exerts a relaxant effect on mouse, rat, and human colonic contraction [9,10]. The relaxant effect is largely through a direct stimulation of ATP-sensitive potassium (K_ATP_) channels, apamin-sensitive small conductance potassium(SK) channels with subsequent hyperpolarization of smooth muscle cells (SMCs) [9,10]. However, the mechanism through which H_2_S exerts its relaxant properties is not fully understood.

Calcium is a fundamental second messenger in SMCs that directly or indirectly controls the contractile activity of smooth muscle [11]. Entry of Ca^2+^ through L-type calcium channels is the primary mechanism for excitation–contraction coupling in gut smooth muscle [12], and L-type calcium channels play a critical role in the amplitude of gut contraction [13]. However, there is no data on whether the relaxant effect of H_2_S on colonic contraction is associated with L-type calcium channels in SMCs. Furthermore, Ca^2+^ influx via the L-type calcium channels plays a central role in intracellular calcium homeostasis [11,14]. Calcium homeostasis in smooth muscle is important for mechanical activity of SMCs, and minor defects in the function of the mechanisms regulating intracellular Ca^2+^ concentration ([Ca^2+^]_i_) can greatly affect the contraction of smooth muscle [14]. It has been reported that H_2_S regulates calcium homeostasis in neurons [15] and cardiomyocytes [16] via L-type calcium channels. Whether H_2_S can also regulate calcium homeostasis in colonic SMCs is still unknown.

Large conductance Ca^2+^-activated K^+^ (BK_Ca_) channel, regarded as one of the main K^+^ channels associated with motility of the colon [17], provides ideal negative feedback regulators in many cell types by decreasing voltage-dependent Ca^2+^ entry through membrane hyperpolarization [17]. One study described an inhibitory effect of H_2_S on BK_Ca_ channels in HEK 293 cells [18]. Another study in rat pituitary tumor cells reported a contradictory result [19]. Whether and how H_2_S interacts with BK_Ca_ channels in colonic SMCs is not clear and warrants in-depth investigation.

Therefore, we hypothesize that H_2_S may regulate the colonic motility by modulating both L-type calcium channels and BK_Ca_ channels in SMCs. In the present study, we investigated the expression of two key enzymes for H_2_S synthesis, and the effect of exogenous H_2_S on spontaneous contraction of colonic muscle strips. In addition, we evaluated the effect of H_2_S on L-type calcium channels and BK_Ca_ channels of single SMCs to determine whether they were involved in mediating the effects of H_2_S.

## Materials and Methods

### Animals

Male Wistar rats weighting 180–200 g were obtained from Vital River (Beijing, China). They were housed in an environmentally controlled room (22±1°C, 65% humidity,12 hour light/dark cycle), and fed standard laboratory chow with free access to water. All animal experimental procedures were approved by the Institutional Animal Care and Use Committee of Wuhan University (Approval ID: WHU 20110312), and adhered to the ethical guidelines of the International Association for the Study of Pain.

### Immunohistochemistry

Immunohistochemical studies were performed on paraffin-embedded, 4-μm-thick sections from proximal colon samples. Following antigen unmasking, sections were incubated overnight at 4°C with rabbit polyclonal anti-CSE and anti-CBS antibodies (1:250 and 1:300, respectively). After being washed twice with PBS, the sections were incubated at room temperature for 2 h in biotinylated anti-mouse or anti-goat secondary antibody and streptavidin-horseradish peroxidase. Diaminobenzidine was used as a chromogen and hematoxylin was used for counterstaining.

### Colonic motility tests in vitro

After rats were killed by cervical dislocation, a 2-cm segment of proximal colon was removed, incised along the mesenteric border, and pinned in a dish with the mucosa facing up. The dish was filled with Tyrode’s buffer (with the following composition in mM: NaCl 147.0, KCl 4.0, CaCl_2_ 2.0, NaH_2_PO_4_ 0.42, Na_2_HPO_4_ 2.0, MgCl_2_ 1.05, glucose 5.5). The circular muscle (CM) or longitudinal muscle (LM) strips(3×10 mm; width×length)were cut along the direction of the circular or longitudinal axis after removing the mucosa and submucosa by sharp dissection. Each fresh smooth muscle strip was fixed in a tissue chamber containing 6 ml Tyrode’s buffer (pH 7.4, bubbled with a mixture of 97% O_2_ and 3%CO_2_).The chamber was maintained at 37°C using a circulating water jacket. One end of the strip was fixed to a hook on the bottom of the chamber, while the other end was attached to an isometric force transducer (JZJOIH, Chengdu, China) to record the contraction. The muscle strips were incubated for 60 min under a resting preload of 1.0 g, washed every 20 min with Tyrode’s buffer. The mean contractile amplitude of colonic strips was recorded on RM6240 multichannel physiological signal system.

### Cell preparation and whole-cell patch-clamp recording

The colonic SMCs were isolated by enzymatic digestion [20]. Strips of colonic muscle were pinned in a Petri dish lined with Sylgard. The mucosa and submucosa were carefully removed under an anatomical microscope. The muscle layer was cut into small segments (2×5 mm), and placed in Ca^2+^-free physiological saline solution (Ca^2+^-free PSS) containing (mM):NaCl 135, KCl 5, glucose10, HEPES 10, MgCl_2_ 1.2 (adjusted PH to 7.4 with NaOH). The segments were incubated for 20–35 min at 36.5°C in digestion medium Ca^2+^-free PSS, containing 0.12%(w/v)collagenaseⅡ,0.2% soybean trypsin inhibitor and 0.2% BSA. After digestion, the supernatant was discarded and the segments were washed 5 times with Ca^2+^-free PSS to remove the enzymes. Single SMCs were dispersed by gentle trituration with a fire-polished Pasteur pipette, and stored at 4°C.

Suspensions of cells were dropped into a perfusion chamber that was mounted with an inverted microscope (Olympus, Japan). After 10 min, the chamber was infused with Tyrode’s buffer (1 ml/min). Currents of L-type calcium channels and BK_Ca_ channels were recorded under a voltage clamp in a standard whole cell configuration using an Axopatch 700B amplifier (Axon Instruments, Burlingham, CA, USA). Acquisition and analysis of the physiological signals were accomplished by pClamp 10.2 (Axon Instruments). The pipette solution for recording L-Ca^2+^ currents (*I*_Ca,L_) contained (mM): CsCl 135, MgCl_2_ 4, HEPES 10, Na_2_ATP 2, EGTA 10, TEA 20 (pH adjusted to 7.3 with CsOH). The pipette solution for recording Ca^2+^-activated K^+^ currents (*I*_BK,Ca_) contained (mM): KCl 125, MgCl_2_ 4, HEPES 10, EGTA 10, Na_2_ATP 5(pH adjusted to 7.3 with KOH). Patch pipettes were made using a micropipette puller(P97;Sutter, USA) and had a resistance of 3–5 MΩ. The data were digitized at 1 kHz, and filtered at 800 Hz. All experiments were conducted at room temperature (approximately 23°C).

### Chemicals

As in numerous previous studies, sodium hydrosulfide (NaHS) was used as a donor for H_2_S. NaHS, tetrodotoxin (TTX), Iberiotoxin (IbTx) and Nifedipine were purchased from Sigma-Aldrich (Sigma-Aldrich Co., St. Louis, MO, USA). NaHS, TTX, and IbTx were dissolved in Tyrode’s buffer. Nifedipine was dissolved in dimethylsulfoxide (DMSO). The final concentration of DMSO was less than 0.03% and had no effect on the cells. The CBS polyclonal antibody was purchased from Santa Cruz Biotechnology (Santa Cruz, CA, USA) and the CSE polyclonal antibody was purchased from Abcam (Abcam (Hong Kong) Ltd., Hong Kong).

### Statistical analysis

The data were analyzed with the pClamp 10.2, SPSS 17.0 and GraphPad Prism 5.01 software. All data in the figures were expressed as the mean ± SD. Significant differences between groups were evaluated using paired Student’s t-tests. Significance level was set at the *P<*0.05.

## Results

### Immunohistochemical localization of CBS and CSE in the proximal colon

**Fig 1:**
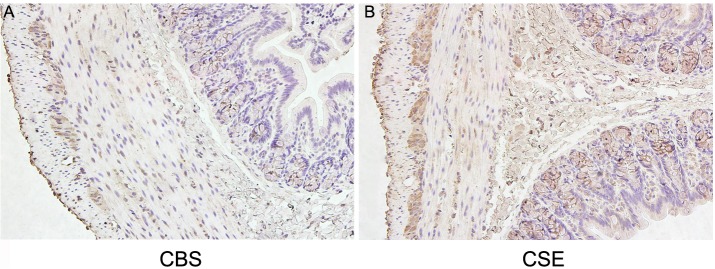
Immunohistochemical localization of CBS (A) and CSE (B) in the rat proximal colon. Magnification 20×.

To determine whether H_2_S can be generated endogenously in the rat proximal colon, the expression of H_2_S synthases was detected using immunohistochemistry. As shown in Fig. 1A, CBS immunoreactivity (IR) in the rat proximal colon was primarily localized in the cytosols of myenteric plexus neurons, although a diffuse pattern was also observed in the epithelial cells and muscular layers. The distribution of CSE was similar to what has been previously reported [2,3], with predominant localization in the cytosol of the circular and longitudinal smooth muscle cells and the nucleus of the myenteric plexus neurons. CSE-IR was also observed in the mucosa and submucosa layers (Fig. 1B).

### The effect of NaHS on contractile activities of colonic strips

**Fig 2:**
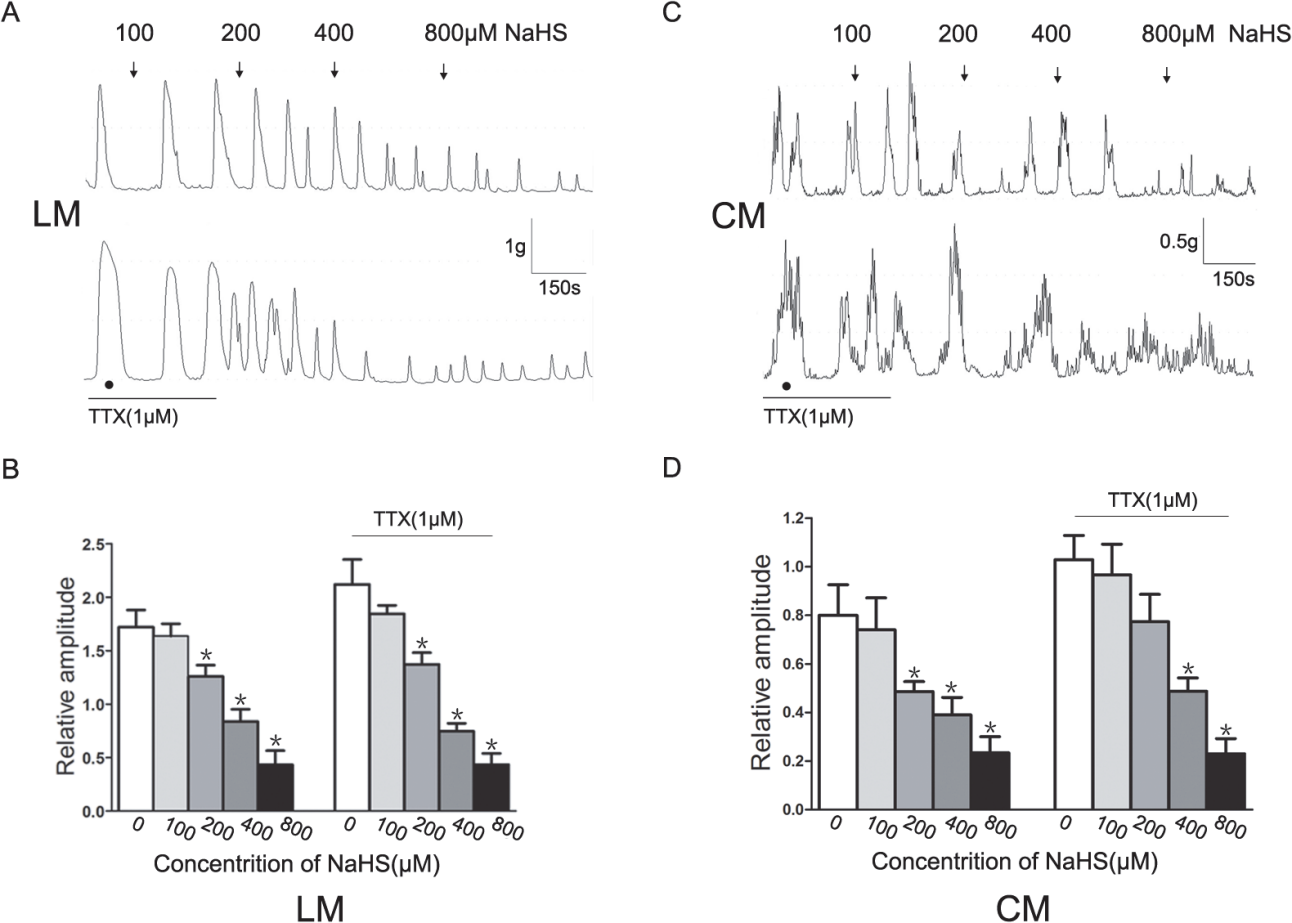
Effect of NaHS on spontaneous contraction of colonic muscle strips. (A and C) NaHS inhibited the spontaneous contractions of longitudinal muscle (LM) and circular muscle (CM) in a concentration-dependent manner, which was still recorded in the presence of TTX (1 μM). (B and D) Summarized results of LM and CM before and after application of NaHS in the presence and absence of TTX. (*n* = 7 for each group, ^*^ *P<*0.05 vs. control)

The H_2_S donor NaHS inhibited the spontaneous contractions of colonic strips in a concentration-dependent manner. As shown in Fig. 2A and C, NaHS significantly reduced the baseline amplitude of spontaneous contractions of LM and CM. The mean amplitude of LM before adding NaHS was 1.72±0.16 g. After the addition of NaHS (100–800 μM), it was reduced to 1.64±0.12 g,1.25±0.09 g,0.84±0.11 g and 0.43±0.13 g, respectively (*P<*0.05 vs. control, Fig. 2B). The mean amplitude of CM before adding NaHS was 0.80±0.13 g, after adding NaHS (100–800 μM), it was reduced to 0.74±0.13 g,0.49±0.04 g,0.39±0.07 g and 0.23±0.07 g, respectively (*P<*0.05 vs. control, Fig. 1D). In the presence of TTX (1 μM), the spontaneous contractions of LM and CM increased, but the inhibitory effects of NaHS were still present.

### NaHS inhibited *I*_Ca,L_ in colonic SMCs

**Fig 3:**
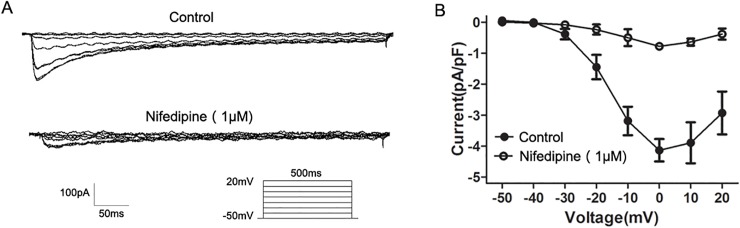
Effect of Nifedipine on *I*_Ca,L_ in colonic SMCs. (A) Original traces of whole cell recordings in response to a series of depolarizing voltage pulses from a holding potential of −50 mV to +20 mV in 10 mV steps before (control) and after application of Nifedipine(1 μM). (B) The representative effects of Nifedipine on the *I-V* relationship of *I*_Ca,L_.

The enzymatically dissociated SMCs appeared elongated. The membrane capacitance of colonic SMCs was 60.8±8.0 pF (*n* = 39). As shown in Fig. 3, *I*_Ca,L_ were elicited by 10 mV depolarizing steps from a constant holding potential of −50 mV to +20 mv for 500 ms using whole-cell voltage-clamp recordings. *I*_Ca,L_ reached the maximal value at approximately 0 mV under control conditions, and this inward current was reduced by 80% with Nifedipine (1 μM) (at 0 mV, *n* = 5, *P<*0.01, vs. control).

**Fig 4:**
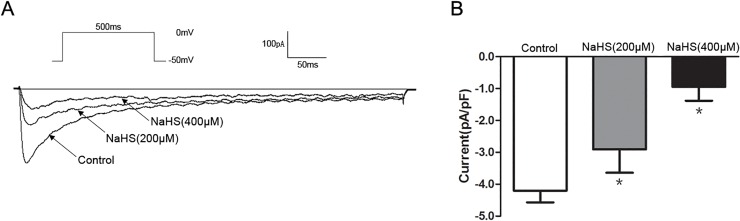
Effect of NaHS on peak *I*_Ca,L_. (A)Representative traces of *I*_Ca,L_ elicited by a single depolarized step pulse from −50 mV to 0 mV before (control) and after application of NaHS(200 and 400 μM). (B) Summarized data showing the density of the currents at 0 mV (*n* = 7 for each group, *P<0.05 vs. control).

Fig. 4A shows the representative traces elicited by a single depolarized step pulse (membrane potential was held at −50 mV and depolarized to 0 mV, 20 s intervals for 500 ms) before (control) and after application of NaHS (200 and 400 μM). Bath application of NaHS (200 and 400 μM) caused a concentration-dependent suppression on the peak of *I*_Ca,L_, the *I*_Ca,L_ density was decreased successively from −4.2±0.37 pA/pF (control) to −2.9±0.73 pA/pF (NaHS 200 μM) and −1.39±0.77 pA/pF (NaHS 400 μM) (*P<*0.05, vs. control, Fig. 4B).

**Fig 5:**
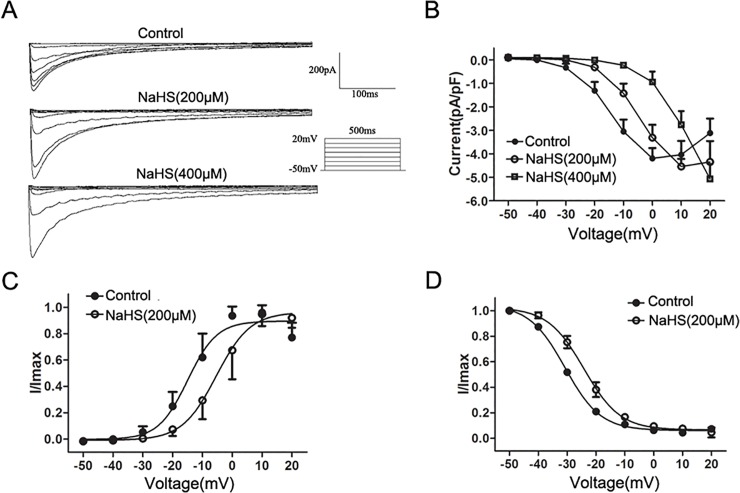
Effect of NaHS on the *I-V* relationship and dynamic characteristics of *I*_Ca,L_. (A) Original traces of whole cell recordings in response to a series of depolarizing voltage pulses from a holding potential of −50 mV to +20 mV in 10 mV steps before (control) and after application of the H_2_S donor NaHS (200 and 400 μM). (B) The representative effects of NaHS (200 and 400 μM) on the *I-V* relationship of *I*_Ca,L_. (C and D) Effect of NaHS(200 μM) on the steady-state activation of *I*_Ca,L_ and the steady-state inactivation of *I*_Ca,L_(*n* = 6 for each group).

**Fig 6:**
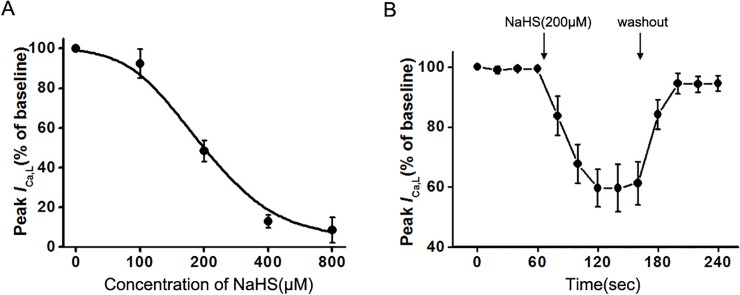
Concentration-dependent property and time course of NaHS on *I*_Ca,L_. (A) A dose response relationship of NaHS-induced inhibition on peak *I*_Ca,L_ at 0 mV. (B) Effect of NaHS(200 μM) on peak *I*_Ca,L_ at 0 mV with a washout period after application of the test compound (*n* = 6 for each group, *P<*0.05 vs. control).

Fig. 5A shows the original traces of whole cell recordings in response to a series of depolarizing voltage pulses from a holding potential of −50 mV to +20 mV in 10 mV steps before (control) and after the application of the H_2_S donor NaHS. NaHS (200 and 400 μM) was successively added to colonic SMCs for 1 min duration per concentration, and the effects were detected. The effects of NaHS on the *I–V* relationship of *I*_Ca,L_ are shown in Fig. 5B. The addition of NaHS (200 and 400 μM) caused an apparent change of the shape of *I–V* curve, and the voltage at which *I*_Ca,L_ reached the maximal value was increased from 0 mV to 10 mV and 20 mV, respectively. The effect of NaHS (200μM) on the steady-state activation of *I*_Ca,L_ was shown in Fig. 5C. The curve was fitted by the Boltzmann equation *G*/*G*_m*ax*_ = 1/[1 + exp(*V_T_* − *V*_1/2_/*κ*)]: *I*/*I*_max_ was used instead of *G*/*G*_max_. *I*/*I*_max_ represents a ratio of currents to the maximum current, and *V*_*T*_ represents the values of the depolarizing pulses with *V*_*1/2*_ representing a half-maximum activation voltage. The H_2_S donor NaHS (200 μM) evoked a significant rightward shift of the *I*_Ca,L_ activation curve, thus the *I*_Ca,L_ *V*_*1/2*_ value was increased from −14.3±1.7 mV to −4.8±2.2 mV (*P<*0.05, vs. control), *κ* values were 4.3±0.8 and 5.0±0.8 in the control and NaHS (200 μM) treated groups (*P*>0.05, vs. control). Fig. 5D shows the effect of NaHS on the steady-state inactivation of *I*_Ca,L_. The curve was fitted by the Boltzmann equation *I*/*I*_max_ = 1/[1 + exp(*V_T_* − *V*_1/2_/*κ*)]: *I*/*I*_max_ represents a ratio of currents to the maximum current of Test pulse, *V*_*T*_ represents the values of the depolarizing potential of the Conditioning pulse, *V*_*1/2*_ represents a half-maximum inactivation voltage. *V*_*1/2*_ values were −30.4±0.6 mV and −24.2±1.0 mV in the control and NaHS (200 μM) treated groups, respectively (*P<*0.05, vs. control), *κ* values were 6.1±0.2 mV and 6.1±0.3 mV in the control and NaHS (200 μM) treated groups, respectively (*P*>0.05, vs. control). NaHS (200 μM) caused a rightward shift in the steady-state inactivation curve of *I*_Ca,L_. At a holding voltage of 0 mV, NaHS (100‒800 μM) decreased the peak of *I*_Ca,L_ in a concentration-dependent manner, with a mean *K*_*d*_ value of 272.8 ± 10.3 μM (Fig. 6A). The dose-response curve was fitted by the logistic function: *Y* = (*A*1 − *A*2)/[1 + (*X*/*X*_0_)^*P*^] + *A*2. Single cells applied with 200 μM NaHS exhibited a time-dependent inhibition of whole-cell *I*_Ca,L_(at 0 mV). The peak of *I*_Ca,L_ decreased successively to 83.72±5.33%,59.71±5.09% and 61.27±5.85% of the value of control at 80,120,160 sec, respectively. The inhibitory effect of NaHS was reversible after washing out NaHS (Fig. 6B).

### The effect of H_2_S donor on BK_Ca_ channels

**Fig 7:**
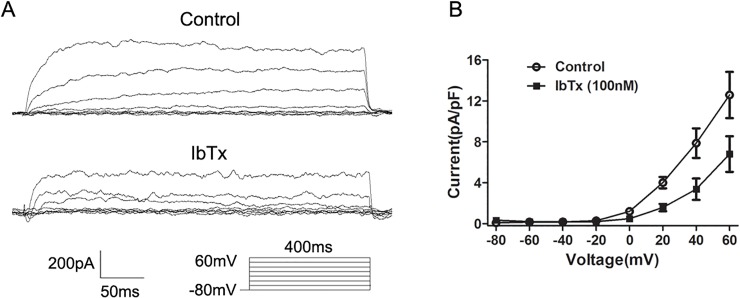
Effect of Iberiotoxin (IbTx) on *I*_BK,Ca_ in colonic SMCs. (A) Original traces of whole cell recordings in response to a series of depolarizing voltage pulses from a holding potential of −80 mV to +60 mV in 20 mV steps before (control) and after the application of IbTx (100 nM). (B) The representative effects of IbTx on the *I-V* relationship of *I*_BK,Ca_.

**Fig 8:**
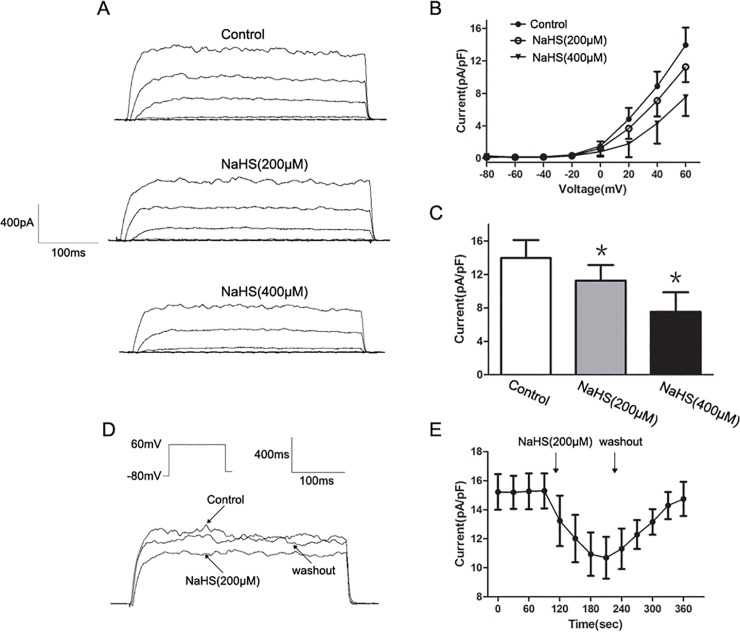
Inhibitory effect of NaHS on *I*_BK,Ca_. (A) Original traces of whole cell recordings in response to a series of depolarizing voltage pulses from a holding potential of −80 mV to +60 mV in 20 mV steps before (control) and after application of NaHS (200 and 400 μM) (B) The representative effects of NaHS at different concentrations on the *I-V* relationship of *I*_BK,Ca_. (C) Summarized data showing the density of the currents at +60 mV. (D and E) Representative traces elicited by a single depolarized step pulse from −80 mV to +60 mV and the time course of NaHS induced inhibition on *I*_BK,Ca_ (*n* = 8 for each group, *P<0.05 vs. control).

To determine the effect of NaHS on *I*_BK,Ca_, *I*_BK,Ca_ was elicited by a series of depolarizing voltage pulses from a holding potential of −80 mV to +60 mV in 20 mV steps. We further characterized the *I*_BK,Ca_ by using IbTx, a specific BK_Ca_ channel blocker [19]. The total outward currents were immediately reduced after the application of IbTx (100 nM) (Fig. 7). Fig. 8A shows the original traces of whole-cell *I*_BK,Ca_ before (control) and after the application of NaHS (200 and 400 μM). The addition of NaHS (200 and 400 μM) caused a significant concentration-dependent decrease on the *I–V* relationship of *I*_BK,Ca_ (Fig. 8B). The *I*_BK,Ca_ density at +60 mV decreased from 14.3±2.2 pA/pF to 11.4±2.1 pA/pF and 7.3±2.5 pA/pF after application of 200 and 400 μM NaHS, respectively (*n* = 8, *P<*0.05 vs. control, Fig. 8C). As shown in Fig. 8D, NaHS (200 μM) significantly decreased the *I*_BK,Ca_ elicited by a single depolarized step pulse (membrane potential was held at −80 mV and depolarized to 60 mV, 30 s intervals for 400 ms, and this effect could be washed out. The effect of NaHS on *I*_BK,Ca_ was found to be time-dependent and reached its maximum during the first minute of application (Fig. 8E).

## Discussion

Hydrogen sulfide is produced in many types of mammalian cells and has been identified as a messenger molecule in the digestive system [1,21,22]. H_2_S has several well-defined physiological effects, including the regulation of GI motility [2,3,21,23, 24]. With few exceptions, the H_2_S donor NaHS inhibits GI motility, causing relaxation of GI smooth muscle [2,3,9,21]. Many of the inhibitory effects of H_2_S on GI motility are mediated directly by ion channels in smooth muscle. However, the role of L-type calcium channels and BK_Ca_ channels in H_2_S-regulated GI motility are unknown. We are the first to report the inhibitory effects of H_2_S on the two ion channels in rat colonic SMCs. Our present study examined the expression of the H_2_S-producing enzymes in the proximal colon and the effects of exogenous H_2_S on L-type calcium channels and BK_Ca_ channels in colonic SMCs. We found that: (1) both CBS and CSE participated in the endogenous production of H_2_S synthesis (2) the H_2_S donor NaHS reversibly inhibited L-type calcium channels and BK_Ca_ channels in SMCs. These data provide the first evidence that H_2_S plays an inhibitory role in the regulation of L-type calcium channels and BK_Ca_ channels, suggesting the involvement of H_2_S in the regulation of calcium homeostasis in smooth muscle of rat colon.

Previously, it has been shown that both CBS and CSE, the enzymes that catalyze the reaction of cysteine to H_2_S, were expressed in colon with marked differences [1–3,8,23,25]. For example, CBS was highly expressed in the lamina propria of rat colon, whereas CSE expression was comparatively low [1]. CSE was evident in neurons of the nervous system and SMCs of rat colon, while CBS was quite diffuse in muscle layers and not expressed in neurons [2]. Another report found that over 90% of human and guinea pig enteric neurons expressed CSE and CBS [8]. Perhaps, these major differences in the distribution of CBS and CSE are due to different technical approaches and species differences. Consistent with the findings of previous studies [3,8], we found that both CBS and CSE were strongly expressed in neurons of the myenteric plexus. CBS-IR and CSE-IR were less intense but still positive in muscle layers. In addition, we found that a strong expression of CSE, but not CBS was also detected in the colonic mucosa. These observations suggest that colonic muscle layers are able to produce H_2_S. GI motility is mainly regulated by the enteric nervous system, and electrical activities of smooth muscle are innervated by hundreds of excitatory and inhibitory motor neurons [26]. CSE or CBS-positive neurons, which can produce H_2_S, can be regarded as inhibitory motor neurons, and H_2_S of neural origin can be considered as a possible neuromodulator. However, H_2_S does not participate in neurally mediated relaxation [2]. Therefore, the role of neurogenic H_2_S needs further study. In the present study, we examined whether enteric neurons were involved in the H_2_S-induced inhibitory effect on colonic LM and CM strips by blocking neural effects with TTX. Consistent with the previous study [9], we found that the H_2_S donor NaHS inhibited the spontaneous contractions of LM and CM strips in a dose-dependent manner, and that the inhibitory effect was not blocked by pretreatment with 1 μM TTX. These results suggest that the inhibitory action of NaHS is exerted directly on SMCs in rat colon.

A number of studies have shown that the relaxant effect of H_2_S on GI smooth muscle is mediated directly via the activation of K^+^ channels located in smooth muscle [9,10,21]. Our previous study also found that glybenclamide, a K_ATP_ channel blocker, significantly reduced the inhibitory effect induced by NaHS [3]. Zhao et al. demonstrated that NaHS inhibited spontaneous contraction of gastric smooth muscle by activating the K_ATP_ channel [21]. Activation of K_ATP_ channel causes efflux of K^+^ and membrane hyperpolarization, resulting in an indirect inhibition of L-type calcium channels. It is known that increasing [Ca^2+^]_i_, which leads to binding to calmodulin and activation of myosin light chain kinase, is the primary stimulus for contraction [14]. Therefore, indirect inhibition of L-type calcium channels accounts in part for the relaxant effect of NaHS on rat colon via the reduction in Ca^2+^ influx and [Ca^2+^]_i_. However, whether the relaxant effect involves direct inhibition of L-type calcium channels is unknown. To investigate this possibility, we examined the effects of NaHS on *I*_Ca,L_. We found that NaHS inhibited the *I*_Ca,L_ in a concentration dependent manner. Furthermore, the shape of *I–V* curve of *I*_Ca,L_ was apparently changed, and the voltage at which *I*_Ca,L_ reached the maximal value was increased, suggesting that the voltage-gated property of L-type calcium channels is modified by NaHS. Moreover, NaHS (200 μM) evoked a significant rightward shift of the steady-state activation curve and a dramatic shift of *V*_*1/2*_ (half-maximum activation voltage) towards higher voltages. A previous study has shown that the actual yield of H_2_S is 33% of the amount of NaHS [27]. As such, NaHS at concentrations of 200 μM may produce 66 μM of H_2_S, which is within the reported physiological range of H_2_S concentration in the brain [28]. Therefore, the inhibitory effect of NaHS on calcium currents at concentration of 200 μM is a physiological effect. In addition, the inhibitory effect of NaHS on peak of *I*_Ca,L_ was dose-dependent from 100 to 800 μM and could be washed out. Taken together, these results infer that exogenous H_2_S directly inhibits the L-type calcium channels that results in a decrease of Ca^2+^ influx and membrane hyperpolarization. These reactions in turn lead to the relaxant effect of the H_2_S donor NaHS on spontaneous contraction of muscle strips in rat colon. In contrast to our findings, a previous study in cardiomyocytes showed that H_2_S inhibited the L-type calcium channel current without changing the channel dynamic characteristics [16]. More recently, in neuronal SH-SY5Y cells, H_2_S increased intracellular calcium via L-type calcium channels [15]. These discrepancies may reflect the diverse characteristics of different cell types. Alternatively, it is possible that an intermediate sensor coupling to the channel, whose nature varies in different cells, may mediate the regulation of L-type Ca^2+^ channels by H_2_S. Future investigations are needed to resolve these questions. We also found that NaHS caused a rightward shift in the steady-state inactivation curve of L-type calcium channels. This indicates that NaHS inhibits the inactivation of L-type calcium channels, and leads to more Ca^2+^ influx through L-type calcium channels. The increase in [Ca^2+^]_i_ could be sufficient to change the membrane potential and elicit a contraction. This apparent contradiction to our previous results may be explained by the finding that BK_Ca_ channels were also inhibited by NaHS in a dose-dependent manner.

BK_Ca_ channels were first studied in SMCs where they are the key players in setting the contractile tone [29]. They are regulated by calcium, as well as by membrane voltage [19,30]. Indeed, because NO and CO are well-known modulators of the BK channels in various cell types including SMCs [31,32], H_2_S might have similar effect on this channel. However, there are only preliminary studies on the effect of H_2_S on BK_Ca_ channels [18,19,33]. The effect of H_2_S on BK_Ca_ channels of colonic SMCs was also detected in our present study. We found that BK_Ca_ channels were significantly inhibited by NaHS, and the addition of NaHS caused a time- and dose-dependent reduction in the *I*_BK,Ca_. The fast decrease of the *I*_BK,Ca_ after the application of NaHS that occurred within seconds and the rapid increase after washout of the drug, favor a direct effect on the channel protein. The results indicate that exogenous H_2_S directly inhibits BK_Ca_ channels in colonic smooth muscle. This same conclusion was reached in a study in HEK 293 cells that stably express human BK_Ca_ α subunit [18]. In contrast to our study however, a recent report showed that NaHS augmented whole-cell BK_Ca_ currents and enhanced single-channel BK_Ca_ activity in rat pituitary tumor cells by increasing channel open probability. Further, the results suggested that the inhibitory effect of NaHS on BK_Ca_ channels might be via BK_Ca_ β4 subunit [19]. This discrepancy may be related to specific BK_Ca_ channel subtypes in different types of cells. Note that the inhibition of H_2_S on BK_Ca_ channels in colonic SMCs indicates that H_2_S has a potentially excitatory effect on colonic motility, while this action of H_2_S was not observed in investigating the effects of H_2_S on the spontaneous contractions of the muscle strips. Maybe the inhibitory effects of H_2_S play a leading role in regulating the colonic motility.

It is known that Ca^2+^ entry through the plasma membrane L-type calcium channels may initiate Ca^2+^ release from ryanodine receptors (RyRs) (Ca^2+^ sparks) in the sarcoplasmic reticulum (SR) [14]. Localized Ca^2+^ transients are because of Ca^2+^ sparks in vascular smooth muscles that can activate BK_Ca_ channels [34] and provide either positive or negative feedback in regulating the excitability of SMCs [14]. Activation of BK_Ca_ channels hyperpolarizes SMCs and reduces Ca^2+^ influx. In the present study, H_2_S inhibited both L-type calcium channels and BK_Ca_ channels, as well as the activation and inactivation of L-type calcium channels. Based on these results, the following hypothesis is proposed to explain the effects of H_2_S on rat colonic SMCs. H_2_S directly inhibits the activation of L-type calcium channels, resulting in a decrease of [Ca^2+^]_i_, which leads to the relaxant effect of the H_2_S donor NaHS on spontaneous contraction of muscle strips. An abnormal decrease in [Ca^2+^]_i_ may provide positive feedback to SR that can store Ca^2+^ and manage its specialized release [14]. RyRs in SR are simulated to release Ca^2+^. Meanwhile, BK_Ca_ channels are inhibited directly by H_2_S, thus causing membrane depolarization and increasing intracellular calcium levels. Both Ca^2+^ released from RyRs and inhibition of BK_Ca_ channels result in a rightward shift of the peak of *I-V* curve and delay of inactivation of L-type calcium channels to maintain calcium homeostasis. It is worth noting that calcium overload leads to altered mitochondrial function [35]. Furthermore, prolonged treatment with H_2_S may induce cell death by increasing cytosolic calcium level [36]. Inhibition of H_2_S at high concentrations on the contraction of colonic muscle strips may due to its toxicological effect. The inhibitory effects of H_2_S on both L-type calcium channels and BK_Ca_ channels suggest that this gaseous molecule plays a role in the regulation of calcium homeostasis in rat colonic SMCs within physiological concentration range. The mechanism by which H_2_S inhibits L-type calcium channels and BK_Ca_ channels in the SMCs membrane is not clear. H_2_S, as a gas, is likely to infiltrate the three-dimensional structures of the channels and may conceivably alter their conformation, and thereby their functions [37]. Our present study also raises a number of interesting questions. For instance, it is unclear why the effects of H_2_S on the two ion channels do not counteract each other. It is not clear whether PKA and PKC/PLC signaling pathways play a part in the H_2_S regulation of calcium homeostasis. Further studies are required to address the possible mechanisms behind H_2_S-related regulation in calcium homeostasis.

In summary, H_2_S inhibits both L-type calcium channels and BK_Ca_ channels in SMCs of rat colon. The relaxant effect of H_2_S on colonic motility is partly due to direct inhibition on L-type calcium channels. These data provide the first evidence that H_2_S may mediate calcium homeostasis in SMCs and therefore play an important role in regulating colonic motility in the rat.
